# 

**Published:** 1883-10

**Authors:** 


					﻿Vol III
OCTOBER, 1883.
NO, 10.
THE
HOMEOPATHIC pHY^lGlAfl,
A MONTHLY JOURNAL OF MEDICAL SCIENCE.
“ Magna est veritas, et prcevalebit.”
IE. X LEM LLI- OD.,
EDITOR.
CONTENTS:
(See inside of Cover.)
PHILADELPHIA:
No. 2109 CHESTNUT STREET.
3ST S3 W YORK:
A. L. CHATTERTON, PUBLISHING COMPANY, PUBLISHER’S AGENTS.
LONDON:
ALFRED HEATH & CO., Sole Agents for Great Britain,
114 Ebury Street, Eaton Square.
Yearly Subscription, $2.50, invariably in advance.
Entered at the Philadelphia Post-Office as Second-Class Mail Matter.
				

